# Health Literacy and Tobacco Use: A Cross-Sectional Study of Associations Among Predominantly Low-Income Adults Motivated to Quit Smoking

**DOI:** 10.3390/healthcare14183105

**Published:** 2026-09-20

**Authors:** Hailey Rueden, Brittany Kelley, Bonnie Douglas, Larry Mason, Jaikanishka Nattamai Subramanian Rajkumar, Puja Raol, Claire A. Spears

**Affiliations:** Department of Health Policy and Behavioral Sciences, School of Public Health, Georgia State University, 140 Decatur St. SE, Atlanta, GA 30303, USA; hrueden1@student.gsu.edu (H.R.); bkelley15@student.gsu.edu (B.K.); bdouglas16@gsu.edu (B.D.); lmason18@student.gsu.edu (L.M.); jnattamaisubramania1@student.gsu.edu (J.N.S.R.); praol1@student.gsu.edu (P.R.)

**Keywords:** health literacy, tobacco use, smoking, sociodemographic characteristics, e-cigarettes

## Abstract

**Background/Objectives:** Tobacco use remains the leading cause of preventable disease, with persistent health disparities for underserved populations. Health literacy may influence tobacco use and cessation. This study examined associations among health literacy, sociodemographic characteristics, and tobacco use behaviors among adults who smoke cigarettes. **Methods:** Baseline data were drawn from a clinical trial among adults who smoked cigarettes and wanted to quit. Participants (*N* = 498) completed the Brief Health Literacy Screener as well as sociodemographic and tobacco use behavior variables. T-tests, chi-square tests, and logistic regression analyses examined associations among health literacy, sociodemographic variables, and tobacco use. **Results:** Compared to participants with adequate health literacy, those with low health literacy were older (*p* < 0.001), had lower socioeconomic status (SES, *p* < 0.001), and were more likely to identify as African American (*p* = 0.005). In bivariate analyses, participants with low health literacy were more likely to smoke within 5 min of waking (*p* = 0.02), indicating higher nicotine dependence. Recent cigar or cigarillo co-use was more common among participants with low health literacy, although not statistically significant (*p* = 0.055). Participants with adequate health literacy were more likely to use electronic vapor products (*p* = 0.03), and this association remained significant after controlling for sociodemographic variables (*p* = 0.03). **Conclusions:** Low health literacy was associated with lower SES, with greater nicotine dependence observed in bivariate analyses. Electronic vapor product use was more common among participants with adequate health literacy. Findings underscore the intersection of health literacy, SES, and broader social determinants of health. Further research is needed to inform health literacy-responsive smoking cessation interventions.

## 1. Introduction

Health literacy, which refers to the degree to which people can access, comprehend, and use health information and services to support informed decisions about their health, is a significant determinant of health outcomes [1,2]. Accordingly, Healthy People 2030 highlights several objectives for increasing health literacy, including a focus on the important role of organizations to support patients’ and consumers’ health literacy [1]. Limited health literacy is a pervasive issue that affects a substantial portion of the adult population, with US national surveys indicating that more than one-third of Americans have basic or below basic health literacy [3,4]. Lower health literacy has been consistently associated with worse health-related outcomes including poorer ability to interpret prescription labels and take medications properly, lower likelihood of preventive healthcare like screening and vaccinations, more hospitalizations, poorer overall health, and higher mortality rates [2].

Moreover, limited health literacy disproportionately affects socioeconomically disadvantaged and marginalized populations, contributing to persistent health disparities [5,6]. Limited health literacy is more common among populations with lower levels of income and education, racially and ethnically minoritized groups, and communities experiencing neighborhood deprivation [3,5,6,7]. Indeed, health literacy is an important mediator of the relationship between socioeconomic status (SES) and numerous health outcomes, including health status, quality of life, health behaviors, and engagement with preventive health services [8]. Limited health literacy further exacerbates health disparities by hindering individuals’ ability to access, understand, and use health information and services, resulting in poorer healthcare access, disease management, and health outcomes, particularly among underserved populations.

Health literacy has also been consistently associated with health risk behaviors that contribute to chronic disease, including tobacco use [2,9,10]. Individuals with limited health literacy may have greater difficulty understanding tobacco-related health information, communicating with healthcare providers, and accessing or utilizing evidence-based smoking cessation treatments, potentially reducing the likelihood of successful quitting [2,11]. Low health literacy has been associated with higher likelihood of current smoking, poorer self-rated health, and higher perceived stress, even after controlling for SES [12,13]. Research indicates that among people who smoke, those with lower health literacy exhibit greater nicotine dependence, have less knowledge about the health risks of smoking, and have lower smoking abstinence self-efficacy [11,14]. In a study among adults who smoked and were trying to quit, those with lower health literacy perceived less social support, which predicted higher depressive symptoms [15]. Research also suggests that lower health literacy predicts lower likelihood of smoking cessation and greater likelihood of relapse [16].

Given the continuously evolving tobacco product market, including e-cigarettes, combustible tobacco products, and other novel tobacco products, there is a need for ongoing evaluation of the associations between health literacy and the use of these other tobacco products. Higher prevalence of co-using cigars and cigarillos along with cigarettes has been documented among lower-SES adults [17], but no known research has examined use of these products by health literacy. A handful of studies have examined associations between health literacy and e-cigarette use, with mixed findings. Using data from the 2016 Behavioral Risk Factor Surveillance System, higher levels of oral health literacy (i.e., greater ease of understanding information communicated orally by healthcare providers) were associated with lower odds of current dual use of both combustible cigarettes and e-cigarettes [18]. However, written health literacy was not associated with tobacco use variables. In a large 2022 study in China, adequate (versus limited) health literacy was associated with lower likelihood of exclusive e-cigarette use, exclusive cigarette use, and dual use [19]. In a large 2020 sample of Taiwanese college students, higher e-health literacy was associated with greater risk perceptions regarding e-cigarettes, indicating that digital information processing might influence how novel tobacco harms are evaluated [20]. In a sample of Jordanian university students, higher health literacy was associated with lower likelihood of tobacco use (e-cigarettes, cigarettes, hookah, or their combination), but the various tobacco products were not examined separately [21]. Research suggests that among adults who smoke combustible cigarettes, complete switching to e-cigarettes may reduce exposure to toxic chemicals, although e-cigarettes are not risk-free [22].

There is a need for additional research to examine associations between health literacy and sociodemographic characteristics with special attention to concurrent tobacco use behaviors among adults who smoke cigarettes. The current study investigated associations among health literacy, sociodemographic characteristics, and tobacco co-use behaviors among predominantly low-SES adults who smoke cigarettes. We hypothesized that lower health literacy would be independently associated with greater nicotine dependence and combustible tobacco co-use, even after controlling for key sociodemographic characteristics.

## 2. Materials and Methods

### 2.1. Procedures

This study involved secondary analysis of baseline data collected from participants who took part in a larger randomized controlled trial (*N* = 504) that aimed to investigate the efficacy of a mindfulness-based text messaging program for smoking cessation [23]. Recruitment targeted a racially and ethnically diverse sample of adults who smoked cigarettes, predominantly individuals with low SES, within the greater Atlanta, Georgia area. Recruitment strategies included flyers, print media, radio, social media, community outreach, and partnerships with local healthcare systems. Interested individuals completed an initial screening via telephone by a trained staff member or an online Qualtrics survey, both of which used identical questions, criteria, and decision rules. After the initial screening, in-person sessions were held at Georgia State University to verify eligibility and obtain informed consent. These baseline sessions were conducted between 2021 and 2024. The study was registered on ClinicalTrials.Gov (NCT04965181) and approved by the Georgia State University Institutional Review Board (Protocol #H20479). All participants provided written consent. The current analysis utilizes baseline data obtained from these participants prior to randomization into the intervention arms of the parent trial.

### 2.2. Participants

Inclusion criteria for the parent study were being at least 18 years of age; currently smoking at least three cigarettes per day (and expired carbon monoxide [CO] > 6 ppm, measured using a Micro+ Smokerlyzer, Bedfont Scientific Ltd., Harrietsham, Maidstone, Kent, UK; distributed in North America by coVita, Santa Barbara, CA, USA); motivated to quit smoking within the next 30 days; having a valid home address in the greater Atlanta area and a functioning telephone number; and the ability to speak, read, and write in English. Exclusion criteria were contraindications to nicotine patch or lozenge; current suicidal ideation; current pregnancy, lactation, or planning pregnancy within the next five months; another household member already enrolled in the study; or prior participation in related trials of mindfulness and/or text messaging-based smoking cessation at Georgia State University.

### 2.3. Measures

Baseline data were collected using a REDCap (Research Electronic Data Capture) survey administered in person. For this secondary analysis, the key measures utilized were:

Health Literacy. Chew et al.’s [24] 3-item Brief Health Literacy Screen is a widely used self-report measure of health literacy. The three items are: “Do you have someone (like a family member, friend, or clinic worker) help you read hospital or clinic materials?”, “Do you have problems learning about your medical condition because of difficulty understanding written information?”, and “How confident are you filling out medical forms by yourself?” (each rated as 0 = “all of the time,” 1 = “most of the time,” 2 = “some of the time,” 3 = “a little of the time,” or 4 = “none of the time”). For this study, scores on the three items were summed (with the latter item on confidence reverse-coded), for a total score with higher numbers indicating higher health literacy. Total scores were then dichotomized to indicate low health literacy (scores 0 through 9) or adequate health literacy (10 or above). This cutoff was based on Haun et al.’s [25,26] classification of inadequate/marginal versus adequate health literacy using the same measure with an additional item; based on the 3-item measure in our study, “inadequate” health literacy corresponds to a score of 0, 1, or 2 on most of the individual items, “marginal” to a score of 3 on most items, and “adequate” to a score of 4 on most items.

Sociodemographic Characteristics. Participants reported their age, sex, level of education, annual household income, number of people supported by that income (to determine poverty status based on the US Census Bureau poverty thresholds [27]), employment status, health insurance status, and race.

Tobacco Use. Participants indicated their average number of cigarettes smoked per day and their time to smoke their first cigarette upon waking (within 5 min, 6 to 30 min, 31 to 60 min, or more than 60 min), both of which comprise the Heaviness of Smoking Index as a validated indicator of nicotine dependence [28]. They were also asked whether their regular brand of cigarettes was menthol or non-menthol and whether they had used various other tobacco products in addition to cigarettes in the past 30 days (yes/no). Definitions and pictures were provided for the following tobacco product categories: “traditional cigars, cigarillos, and filtered cigars” (with common brands presented for each of these products), “hookah” (described as a type of water pipe, sometimes also called a ‘narghile’ pipe, often smoked in groups at cafes or in hookah bars); “chewing tobacco, dip or snuff, snus, or dissolvable tobacco;” and “electronic vapor products” (“such as e-cigarettes, e-cigars, e-hookahs, e-pipes, vape pens, hookah pens, pod and pod mod vaporizers, and personal vaporizers/mods; these devices are battery-powered and contain a liquid that usually contains nicotine and is vaporized and inhaled,” with some common brands provided). A separate question also asked about use of electronic vapor products (EVPs) in the past 7 days.

### 2.4. Data Analysis

The secondary analysis utilized an analytic sample of N=498 derived from the parent trial’s total enrollment of N=504 after excluding 6 participants with missing health literacy data. *t*-tests and chi-square tests examined associations between health literacy (dichotomized to indicate low vs. adequate health literacy) and sociodemographic variables and smoking behaviors. Next, for the tobacco use variables that were significantly related to health literacy, logistic regression analyses predicted tobacco use from health literacy, controlling for sociodemographic variables that were significantly related to health literacy. Several categories within the employment, health insurance, and race variables had small cell sizes, resulting in large standard errors and unstable regression estimates. Therefore, selected categories were combined to address sparse data and improve the precision, stability, and interpretability of the regression models. Variance inflation factor (VIF) values were examined to assess potential multicollinearity among the independent variables. The VIF values ranged from 1.03 to 1.28, indicating that multicollinearity was not a concern. For all analyses, missing data were addressed using listwise deletion, and corresponding valid sample sizes are reported. Data were analyzed using IBM SPSS Statistics (version 31).

## 3. Results

### 3.1. Sample Characteristics

Of the 504 participants enrolled in the parent trial, 6 had missing health literacy scores, resulting in an analytic sample of 498 (123 with low health literacy and 375 with adequate health literacy). Sociodemographic characteristics for the full sample and by health literacy level are shown in Table 1. Overall, participants had a mean age of 50.3 (SD 12.7), 60.2% were male, and 38.8% had annual household incomes below the federal poverty level.

### 3.2. Associations Between Health Literacy and Sociodemographic Variables

Compared to participants with adequate health literacy, those with low health literacy tended to be older (median age 57 vs. 50, *t* (496) = 3.40, *p* < 0.001) and have lower levels of education (22.8% of those with low health literacy had less than high school education vs. 7.5% of those with adequate health literacy, χ^2^ (5) = 36.31, *p* < 0.001). Participants with low health literacy were also more likely to be living below the US federal poverty level (54.5% of those with low health literacy vs. 33.7% of those with adequate health literacy, χ^2^ (1) = 16.75, *p* < 0.001), less likely to be working full-time (15.4% vs. 40.6%, χ^2^ (1) = 17.92, *p* < 0.001, χ^2^ (8) = 43.78, *p* < 0.001), and less likely to have health insurance through an employer (13.0% vs. 26.5%, χ^2^ (6) = 18.65, *p* = 0.005). Participants with low health literacy were more likely to identify as Black or African American (76.4% of those with low vs. 60.8% of those with adequate health literacy) and less likely to be White (14.6% of those with low vs. 27.2% of those with adequate health literacy), χ^2^ (5) = 16.66, *p* = 0.005. Health literacy was not associated with sex (*p* = 0.69).

### 3.3. Associations Between Health Literacy and Tobacco Use Behaviors

Tobacco use variables for the full sample and by health literacy level are shown in Table 2. On average, participants smoked 14.7 cigarettes per day at baseline, with no significant differences by health literacy. Participants with lower health literacy were more likely to smoke within 5 min of waking (indicating higher nicotine dependence, 46.3% vs. 34.9%), χ^2^ (1) = 5.11, *p* = 0.024. Recent cigar or cigarillo co-use was more common among those with low health literacy than those with adequate health literacy (36.4% [44/121] vs. 27.2% [101/371]), although the difference was not statistically significant, $\chi^{2}\left(1\right)=3.67,\;p=0.055$. On the other hand, participants with adequate health literacy were more likely to report recent use of electronic vapor products (EVPs; past 30-day use of EVPs: 25.5% of those with adequate health literacy vs. 15.7% of those with low health literacy, χ^2^ (1) = 4.90, *p* = 0.027; past 7-day use of EVPs: 17.3% of those with adequate vs. 9.1% of those with low health literacy, χ^2^ (1) = 4.79, *p* = 0.029). Health literacy level was not associated with the number of cigarettes per day, preference for menthol cigarettes, or recent use of hookah or chewing tobacco. Prevalence rates for recent hookah and chewing tobacco use were relatively low, resulting in small cell sizes.

### 3.4. Associations Between Health Literacy and Tobacco Use Behaviors, Controlling for Sociodemographic Variables

Table 3 shows results of the logistic regression models predicting tobacco use variables from health literacy and sociodemographic covariates, including both the crude odds ratios (CORs) and adjusted odds ratios (AORs) for each predictor. In the logistic regression analyses predicting each of the above tobacco use variables that differed by health literacy (time to first cigarette; past 30-day use of cigars or cigarillos; past 30-day and 7-day use of EVPs) and controlling for age, education, poverty status, employment status, health insurance status, and race, health literacy remained significant in the model predicting past 7-day use of EVPs, *Wald* (1) = 4.51, *p* = 0.034. Participants with adequate health literacy were about twice as likely (AOR = 2.26, 95% CI 1.07, 4.80) to report using EVPs (in addition to combustible cigarettes) after controlling for age, race, and several indicators of SES. In that model, age (*Wald* (1) = 19,90, *p* < 0.001) and poverty status (*Wald* (1) = 7.67, *p* = 0.006) were also significant predictors. Older participants were less likely to report past 7-day use of EVPs than younger participants (AOR = 0.94, 95% CI 0.92, 0.97). On the other hand, participants living below the poverty level were more likely to report past 7-day use of EVPs (40 of 186, 21.5%) than those living at or above the poverty level (35 of 301, 11.6%).

In predicting past-30-day use of EVPs, age (*Wald* (1) = 31.40, *p* < 0.001) and poverty status (*Wald* (1) = 6.78, *p* = 0.009) were significant. Older participants were less likely to have used EVPs in the past 30 days (AOR = 0.94, 95% CI 0.91, 0.96). However, participants living in poverty were more likely to use EVPs (50 of 186, 26.9%) than those at or above the poverty level (63 of 301, 20.9%). Health literacy was no longer significant (*p* = 0.19) after controlling for age, race, and indicators of SES.

In predicting time to first cigarette, only education (*Wald* (5) = 16.32, *p* = 0.006) was significant. Participants with lower education were more likely to smoke their first cigarette within 5 min of waking (79 of 155, 51.0% of participants with high school, GED, or less education vs. 108 of 339, 31.9% of those with more than high school education). Health literacy was no longer significant (*p* = 0.53) after controlling for education and other sociodemographic variables.

In predicting past-30-day use of cigars and cigarillos, age (*Wald* (1) = 18.02, *p* < 0.001), race (*Wald* (2) = 14.10, *p* < 0.001), poverty status (*Wald* (1) = 4.83, *p* =0.028), and health insurance status (*Wald* (2) = 7.78, *p* = 0.02), but not health literacy (*p* = 0.11), were significant. Older participants were less likely to use cigars/cigarillos (OR = 0.95, 95% CI 0.93, 0.97). However, African Americans (108 of 319, 33.9%) and people identifying with other racial groups (18 of 56, 32.1%) were more likely to use cigars/cigarillos than white participants (20 of 102, 16.4%). Participants living below the federal poverty line (73 of 191, 38.2%) were more likely to use cigars/cigarillos than those at or above the poverty level (71 of 303, 23.4%). Finally, participants without health insurance coverage (45 of 107, 42.1%) were more likely to use cigars/cigarillos than those with private health insurance (23 of 116, 19.8%).

## 4. Discussion

This study examined sociodemographic correlates of health literacy and associations between health literacy and tobacco use among adults who smoked cigarettes and were attempting to quit. Even within a relatively low-SES sample, there were notable differences in both sociodemographic variables and tobacco use by health literacy. Participants with lower health literacy experienced substantially greater social disadvantage across multiple domains. Participants with low health literacy also demonstrated higher nicotine dependence. Recent cigar or cigarillo co-use was more common among participants with low health literacy, although not statistically significant. On the other hand, participants with high health literacy were more likely to use electronic vapor products (e.g., e-cigarettes), even after controlling for other sociodemographic variables. These findings highlight the complex relationships among health literacy, sociodemographic characteristics, and tobacco use, and underscore the importance of developing tobacco control and cessation strategies that address the needs of adults with low health literacy, who may experience multiple, intersecting forms of disadvantage.

Among this sample of adults who smoked cigarettes and were motivated to quit, individuals with low health literacy were older, had lower educational attainment, were more likely to live below the poverty level, less likely to work full-time, and less likely to have employer-sponsored insurance. These findings are consistent with the previous literature showing that health literacy is strongly linked to lower SES [3,4,5,6,7,29]. Importantly, these differences emerged despite the overall sample already having relatively low SES. Participants with low health literacy were also disproportionately Black or African American, aligning with extant research [4,6,29]. Framed within an intersectionality perspective, health literacy exists within a broader social context shaped by structural racism, educational inequities, and economic disadvantage [29,30,31,32].

Although not statistically significant (*p* = 0.055), recent cigar or cigarillo co-use was more common among participants with low health literacy than among those with adequate health literacy in bivariate analyses. This may nevertheless have clinical relevance, given that concurrent use of cigarettes and cigars may increase exposure to harmful toxicants and carcinogens beyond that associated with cigarette smoking alone, potentially increasing tobacco-related health risks. Prior research suggests that adults who smoked cigarettes and also used other tobacco products smoked at least as many cigarettes per day and had mortality risks comparable to, and in some cases exceeding, those of individuals who smoked cigarettes exclusively [33]. In the present study, the association between health literacy and cigar or cigarillo co-use was attenuated after adjustment for other sociodemographic variables, suggesting that differences in co-use may reflect the broader social and structural factors associated with low health literacy rather than health literacy alone. This interpretation is consistent with extant work indicating that among U.S. adults who smoke cigarettes, those living in poverty and racial/ethnic communities were disproportionately likely to also use little cigars, cigarillos, or filtered cigars [17]. Larger, adequately powered studies are needed to determine whether the observed difference represents a meaningful and reproducible association and to clarify whether health literacy-responsive approaches may have a role in addressing co-use among potentially higher-risk groups.

On the other hand, among this sample of adults who smoked cigarettes and were trying to quit, those with low health literacy were less likely to use EVPs (e.g., e-cigarettes), and the association between low health literacy and lower likelihood of past-7-day EVP use remained significant after adjusting for multiple indicators of SES. Although e-cigarettes are not risk-free, they may be useful for harm reduction if people are able to substitute combustible cigarettes with a lower-risk alternative in the process of quitting smoking. Among adults who smoke cigarettes, e-cigarette use has been associated with reductions in combustible cigarette smoking [34]. Although evidence is somewhat mixed on the effects of e-cigarette use for smoking cessation, meta-analyses support the use of e-cigarettes containing nicotine to increase quit rates compared to nicotine replacement therapy [35]. It is possible that people with low health literacy have lower awareness of EVPs and their relative risks, may have less access to cessation messaging involving e-cigarettes, or may be less likely to discuss e-cigarettes with healthcare providers. It is also possible that financial or access barriers limit the use of e-cigarettes among people with low health literacy, although poverty actually predicted greater e-cigarette use, making this relationship more nuanced. Because dual use is common and exposes users to both cigarettes and potential e-cigarette toxicants [36], these findings should not be interpreted as suggesting that increased e-cigarette uptake is universally desirable. Rather, they may indicate differences in access to or understanding of emerging nicotine products and harm reduction information. Ongoing work is needed to educate the public, including people with lower levels of health literacy, about the relative risks of e-cigarettes. If evidence continues to support the use of e-cigarettes for smoking cessation, it will be important to communicate optimal ways to completely switch to e-cigarettes and eventually stop using all tobacco and nicotine products.

### 4.1. Public Health Implications

There is a need for tobacco cessation interventions to incorporate health literacy-responsive communication strategies, including the use of plain language, teach-back techniques, and accessible educational materials [37]. Public health messaging about emerging nicotine products, including e-cigarettes, should be clear, accessible, and tailored to individuals with low health literacy levels. This is particularly important given the evolving evidence on the risks and potential harm reduction benefits of these products. In addition, tobacco control efforts should consider health literacy within the broader context of social determinants of health. Given the clustering of low health literacy with poverty, lower educational attainment, and other indicators of social disadvantage observed in this study, interventions that address health literacy should complement, rather than replace, efforts to address the root causes of health disparities [8]. Reducing tobacco-related disparities will require multilevel interventions that address not only individual knowledge and communication needs, but also the structural and societal factors (including educational inequities, economic disadvantage, barriers to healthcare access, and systemic racism) that shape tobacco use behaviors and opportunities for cessation [38].

### 4.2. Limitations, Strengths, and Future Directions

This study is limited by reliance on cross-sectional data, precluding the ability to establish causal relationships. All measures were self-reported, and health literacy was based on the 3-item Brief Health Literacy Screen [24], which assesses perceived difficulty understanding written health information, need for assistance with written materials, and confidence completing medical forms. This measure primarily captures reading-related difficulties, does not include objective assessment, and does not assess other aspects of health literacy, such as the ability to communicate with healthcare providers, navigate healthcare systems, or evaluate online health information. The use of self-reported measures in this study may also introduce reporting and recall bias. Additionally, listwise deletion in multivariable models may introduce selection bias when missingness across covariates is systematically related to the outcome or other characteristics of the study population.

Furthermore, because the sample primarily comprised African American, lower-SES adults seeking to quit smoking within a single metropolitan area, findings may have limited generalizability to other populations. Additionally, data regarding other potential confounders—such as participant alcohol and substance use history, housing stability, mental health diagnoses and psychological distress, etc.—were not included in this secondary analysis and warrant examination in future research. Certain tobacco product categories (such as hookah and chewing tobacco) had very small cell sizes, leading to unstable estimates. Nonetheless, the study is strengthened by a relatively large sample, focus on an underserved population, and examination of multiple tobacco products, adding to the scant literature on associations between health literacy and tobacco products beyond traditional cigarettes.

This study is limited by the use of cross-sectional baseline data. It is possible that tobacco cessation interventions influence health literacy and tobacco co-use patterns over time. Future longitudinal research should explore how these variables evolve throughout the course of smoking cessation treatment. Longitudinal studies are needed to better understand the mechanisms through which health literacy influences tobacco product use and cessation behaviors, as well as whether health literacy moderates response to tobacco cessation interventions. These studies could utilize a mixed-method approach, combining quantitative assessments of health literacy with interviews to better understand the experiences of individuals navigating the healthcare system with varying levels of health literacy. Future research should also move beyond examining sociodemographic characteristics as independent predictors and instead adopt intersectional approaches that recognize the interconnected ways in which health literacy, race, SES, age, and other social determinants shape tobacco use behaviors and contribute to tobacco-related health disparities.

## 5. Conclusions

Low health literacy was associated with greater social disadvantage and distinct patterns of tobacco product use among adults who smoked and were trying to quit. Tobacco control efforts should incorporate health literacy-responsive communication while also addressing the social and structural conditions that contribute to disparities in tobacco use and cessation.

## Figures and Tables

**Table 1:** Sociodemographic variables by health literacy level.

	Total (*N* = 498)	Low Health Literacy (*N* = 123)	Adequate Health Literacy (*N* = 375)	*p*
**Mean Health Literacy Score (SD)**	10.7 (1.9)	7.7 (1.2)	11.7 (0.6)	<0.001
Median Health Literacy Score	12	8	12	
Range of Health Literacy Scores	4 to 12	4 to 9	10 to 12	
**Mean age in years (SD)**	50.3 (12.7)	53.6 (12.34)	49.2 (12.7)	<0.001
Median age	52	57	50	
**Sex—no. (%)**				0.686
Male	300 (60.2)	76 (61.8%)	224 (59.7%)	
Female	198 (39.8)	47 (38.2)	151 (40.3)	
**Education—no. (valid %)**				<0.001
Less than high school	56 (11.3)	28 (22.8)	28 (7.5)	
High school or GED	99 (19.9)	33 (26.8)	66 (17.6)	
Some college or technical school	165 (33.2)	38 (30.9)	127 (34.0)	
Associate’s Degree	61 (12.3)	8 (6.5)	53 (14.2)	
Bachelor’s Degree	69 (13.9)	10 (8.1)	59 (15.8)	
Graduate studies or graduate degree	47 (9.5)	6 (4.9)	41 (11.0)	
Missing	1	0	1	
**Poverty Status—no. (valid %)**				<0.001
Below US federal poverty level	192 (38.8)	66 (54.5)	126 (33.7)	
At or above poverty level	303 (61.2)	55 (45.5)	248 (66.3)	
Missing	3	2	1	
**Employment Status—no. (valid %)**				<0.001
Regular full-time work	171 (34.3)	19 (15.4)	152 (40.6)	
Regular part-time work	67 (13.5)	18 (14.6)	49 (13.1)	
Unemployed, looking for work	43 (8.7)	12 (9.8)	31 (8.3)	
Unemployed, not looking for work	17 (3.4)	8 (6.5)	9 (2.4)	
Homemaker	6 (1.2)	1 (0.8)	5 (1.3)	
Student	21 (4.2)	1 (0.8)	20 (5.3)	
Retired	73 (14.7)	27 (22.0)	46 (12.3)	
Unable to work or disabled	81 (16.3)	33 (26.8)	48 (12.8)	
Other	18 (3.6)	4 (3.3)	14 (3.7)	
Missing	1	0	1	
**Health Insurance Status—no. (valid %)**				0.005
Health insurance through employer	115 (23.1)	16 (13.0)	99 (26.5)	
Medicare	87 (17.5)	29 (23.6)	58 (15.5)	
Medicaid	80 (16.1)	28 (22.8)	52 (13.9)	
Federal health insurance marketplace	57 (11.5)	11 (8.9)	46 (12.3)	
VA, DOD, or other military programs	21 (4.2)	7 (5.7)	14 (3.7)	
Health insurance from another source	28 (5.6)	4 (3.3)	24 (6.4)	
No health insurance	109 (21.9)	28 (22.8)	81 (21.6)	
Missing	1	0	1	
**Race—no. (%)**				0.005
White	120 (24.1)	18 (14.6%)	102 (27.2)	
Black or African American	322 (64.7)	94 (76.4)	228 (60.8)	
Asian	11 (2.2)	0	11 (2.9)	
American Indian/Alaska Native	1 (0.2)	0	1 (0.3)	
More than one race	26 (5.2)	4 (3.3)	22 (5.9)	
Other	18 (3.6)	7 (5.7)	11 (2.9)	

**Notes.** Reported *p* values correspond to *t* tests (for health literacy scores and age) and chi square tests (for all other variables) comparing sociodemographic variables for people with low versus adequate health literacy. Health literacy was assessed with Chew et al.’s [24] health literacy measure and dichotomized to indicate low health literacy (scores 0 through 9) or adequate health literacy (10 or above). For the race category, participants who selected ‘Other’ were prompted to provide a free-text response.

**Table 2:** Tobacco use behaviors by health literacy level.

	Total (*N* = 498)	Low Health Literacy (*N* = 123)	Adequate Health Literacy (*N* = 375)	*p*
**Mean Cigarettes per Day (SD)**	14.7 (8.2)	15.43 (8.53)	14.46 (8.12)	0.270
Median Cigarettes per Day	13	15	12	
**Time to first cigarette—no. (valid %)**				0.024
Within 5 min of waking	187 (37.8)	57 (46.3)	130 (34.9)	
More than 5 min of waking	308 (62.2)	66 (53.7)	242 (65.1)	
Missing	3	0	3	
**Menthol as regular cigarette brand—no. (valid %)**				0.211
Menthol	354 (71.8)	93 (76.2)	261 (70.4)	
Non-Menthol	139 (28.2)	29 (23.8)	110 (29.6)	
Missing	5	1	4	
**Cigars or Cigarillos—no. (valid %)**				0.055
Past-30-day use				
Yes	145 (29.5)	44 (36.4)	101 (27.2)	
No	347 (70.5)	77 (63.6)	270 (72.8)	
Missing	6	2	4	
**Hookah—no. (valid %)**				0.075
Past-30-day use				
Yes	28 (5.7)	3 (2.5)	25 (6.8)	
No	459 (94.3)	118 (97.5)	341 (93.2)	
Missing	11	2	9	
**Chewing tobacco—no. (valid %)**				0.126
Past-30-day use				
Yes	7 (1.4)	0	7 (1.9)	
No	486 (98.6)	122 (100)	364 (98.1)	
**Electronic vapor products (e.g., e-cigarettes)—no. (valid %)**				
**Past 30-day use**				0.027
Yes	113 (23.1)	19 (15.7)	94 (25.5)	
No	377 (76.9)	102 (84.3)	275 (74.5)	
Missing	8	2	6	
**Past 7-day use**				0.029
Yes	75 (15.3)	11 (9.1)	64 (17.3)	
No	415 (84.7)	110 (90.9)	305 (82.7)	
Missing	8	2	6	

**Notes.** Reported *p* values correspond to *t* tests (for cigarettes per day) and chi square tests (for all other variables) comparing tobacco use variables for people with low versus adequate health literacy. Health literacy was assessed with Chew et al.’s [24] health literacy measure and dichotomized to indicate low (scores 0–9) or adequate health literacy (10 or above).

**Table 3:** Logistic regression models predicting tobacco use variables from health literacy and sociodemographic covariates.

**Past 7-Day Use of Electronic Vapor Products**				
**Predictor Variable**	**COR (95% CI)**	***p***	**AOR (95% CI)**	***p***
Health Literacy				
Adequate vs. low health literacy (ref)	2.08 (1.06, 4.09)	0.03	2.26 (1.07, 4.80)	0.03
Age	0.95 (0.93, 0.97)	<0.001	0.94 (0.92, 0.97)	<0.001
Poverty Status				
Living in poverty vs. not (ref)	2.01 (1.23, 3.29)	0.005	2.46 (1.30, 4.65)	0.006
Education	--	0.85	--	0.73
Less than high school (ref)	1.00		1.00	
High school or GED	1.42 (0.52, 3.90)	0.50	1.86 (0.61, 5.55)	0.28
Some college or technical school	1.75 (0.69, 4.48)	0.24	2.04 (0.72, 5.81)	0.18
Associate’s Degree	1.36 (0.45, 4.10)	0.59	1.38 (0.40, 4.78)	0.62
Bachelor’s Degree	1.20 (0.40, 3.60)	0.75	1.59 (0.45, 5.60)	0.47
Graduate studies or graduate degree	1.31 (0.41, 4.19)	0.65	1.21 (0.30, 4.86)	0.79
Employment Status	--	0.04	--	0.27
Full-time (Ref)	1.00		1.00	
Part-time	2.26 (1.09, 4.66)	0.03	1.90	0.15
Unemployed	1.47 (0.65, 3.34)	0.36	1.34	0.59
Retired	0.40 (0.13, 1.22)	0.11	1.14	0.85
Unable to work or Disabled	1.74 (0.85, 3.54)	0.13	2.39	0.08
Other	1.71 (0.73, 4.05)	0.22	0.74	0.56
Health Insurance Status	--	0.27	--	0.68
Private Insurance (ref.)	1.00		1.00	
Government Insurance	1.73 (0.88, 3.41)	0.11	1.45 (0.60, 3.46)	0.41
No Health Insurance	1.70 (0.78, 3.72)	0.18	1.19 (0.47, 3.03)	0.72
Race	--	0.02	--	0.17
White (ref)	1.00		1.00	
African American	0.56 (0.32, 0.98)	0.04	0.59 (0.30, 1.16)	0.12
Other	1.33 (0.63, 2.82)	0.46	1.17 (0.50, 2.71)	0.72
**Past 30-Day Use of Electronic Vapor Products**				
**Predictor Variable**	**COR (95% CI)**	***p***	**AOR (95% CI)**	***p***
Health Literacy				
Adequate vs. low health literacy (ref)	1.82 (1.06, 3.13)	0.03	1.52 (0.81, 2.83)	0.19
Age	0.94 (0.92, 0.95)	<0.001	0.94 (0.91, 0.96)	<0.001
Poverty Status				
Living in poverty vs. not (ref)	1.33 (0.87, 2.04)	0.19	2.15 (1.21, 3.83)	0.009
Education		0.63		0.33
Less than high school (ref)	1.00		1.00	0.32
High school or GED	1.63 (0.67, 3.96)	0.283	1.65 (0.62, 4.39)	0.16
Some college or technical school	1.94 (0.85, 4.44)	0.119	1.93 (0.77, 4.84)	0.67
Associate’s Degree	1.52 (0.58, 4.02)	0.395	1.27 (0.42, 3.81)	0.09
Bachelor’s Degree	2.18 (0.87, 5.47)	0.097	2.51 (0.88, 7.22)	0.96
Graduate studies or graduate degree	1.63 (0.59, 4.46)	0.343	1.03 (0.31, 3.46)	0.315
Employment Status		0.01		0.50
Full-time (Ref)	1.00		1.00	
Part-time	1.33 (0.71, 2.47)	0.37	1.01 (0.47, 2.15)	0.98
Unemployed	0.74 (0.36, 1.53)	0.42	0.55 (0.22, 1.37)	0.20
Retired	0.21 (0.08, 0.54)	0.001	0.58 (0.179, 1.89)	0.37
Unable to work or Disabled	0.81 (0.43, 1.52)	0.51	1.11 (0.46, 2.65)	0.82
Other	1.38 (0.68, 2.78)	0.38	0.62 (0.26, 1.48)	0.28
Health Insurance Status	--	0.32	--	0.36
Private Insurance (ref.)	1.00		1.00	
Government Insurance	1.06 (0.62, 1.81)	0.83	1.62 (0.78, 3.34)	0.20
No Health Insurance	1.51 (0.82, 2.79)	0.19	1.67 (0.78, 3.58)	0.19
Race	--	<0.001	--	0.14
White (ref)	1.00		1.00	
African American	0.45 (0.28, 0.72)	0.001	0.57 (0.32, 1.02)	0.06
Other	1.24 (0.64, 2.40)	0.52	0.92 (0.44, 1.93)	0.82
**Time to First Cigarette**				
**Predictor Variable**	**COR (95% CI)**	***p***	**AOR (95% CI)**	***p***
Health Literacy	0.64 (0.42, 0.97)	0.03	0.86 (0.54, 1.37)	0.53
Adequate vs. low health literacy (ref)				
Age	1.00 (0.99, 1.01)	0.99	0.99 (0.97, 1.01)	0.44
Poverty Status				
Living in poverty vs. not (ref)	1.85 (1.28, 2.69)	0.001	1.34 (0.85, 2.13)	0.21
Education		<0.001		0.006
Less than high school (ref)	1.00		1.00	
High school or GED	0.68 (0.35, 1.31)	0.25	0.79 (0.39, 1.59)	0.51
Some college or technical school	0.48 (0.26, 0.88)	0.03	0.51 (0.26, 1.00)	0.05
Associate’s Degree	0.27 (0.12, 0.58)	<0.001	0.32 (0.14, 0.73)	0.007
Bachelor’s Degree	0.21(0.10, 0.46)	<0.001	0.26 (0.11, 0.60)	0.002
Graduate studies or graduate degree	0.30 (0.13, 0.68)	0.004	0.36 (0.14, 0.88)	0.03
Employment Status	--	0.15	--	0.92
Full-time (Ref)	1.00		1.00	
Part-time	1.78 (0.99, 3.19)	0.05	1.24 (0.64, 2.40)	0.53
Unemployed	1.78 (0.97, 3.27)	0.06	1.16 (0.54, 2.45)	0.71
Retired	1.50 (0.85, 2.66)	0.16	1.00 (0.46, 2.18)	0.99
Unable to work or Disabled	1.92 (1.11, 3.31)	0.02	0.89 (0.43, 1.84)	0.76
Other	1.24 (0.62, 2.48)	0.53	0.82 (0.37, 1.83)	0.63
Health Insurance Status	--	0.003	--	0.07
Private Insurance (ref.)	1.00		1.00	
Government Insurance	2.33 (1.43, 3.79)	<0.001	1.86 (1.00, 3.47)	0.05
No Health Insurance	1.78 (1.00, 3.17)	0.05	1.15 (0.58, 2.25)	0.70
Race	--	0.70	--	0.50
White (ref)	1.00		1.00	
African American	1.11 (0.72, 1.71)	0.64	0.75 (0.45, 1.23)	0.25
Other	0.88 (0.45, 1.70)	0.69	0.78 (0.38, 1.60)	0.49
**Past 30-Day Use of Cigars or Cigarillos**				
**Predictor Variable**	**COR (95% CI)**	***p***	**AOR (95% CI)**	***p***
Health Literacy				
Adequate vs. low health literacy (ref)	0.67 (0.43, 1.02)	0.06	0.66 (0.40, 1.09)	0.11
Age	0.97 (0.96, 0.99)	<0.001	0.96 (0.94, 0.98)	<0.001
Poverty Status				
Living in poverty vs. not (ref)	2.02 (1.36, 3.00)	<0.001	1.78 (1.06, 2.98)	0.028
Education		0.04		0.57
Less than high school (Ref)	1.00		1.00	
High school or GED	0.89 (0.45, 1.75)	0.73	0.99 (0.47, 2.09)	0.97
Some college or technical school	0.60 (0.32, 1.13)	0.11	0.74 (0.36, 1.52)	0.41
Associate’s Degree	0.57 (0.26, 1.23)	0.15	0.90 (0.38, 2.15)	0.81
Bachelor’s Degree	0.30 (0.13, 0.68)	0.004	0.49 (0.19, 1.25)	0.13
Graduate studies or graduate degree	0.49 (0.21, 1.13)	0.10	0.99 (0.37, 2.67)	0.98
Employment Status	--	0.10	--	0.39
Full-time (Ref)	1.00		1.00	
Part-time	1.22 (0.66, 2.24)	0.52	0.70 (0.33, 1.46)	0.34
Unemployed	1.49 (0.80, 2.78)	0.21	0.64 (0.29, 1.45)	0.29
Retired	0.52 (0.26, 1.03)	0.06	0.52 (0.21, 1.33)	0.17
Unable to work or Disabled	1.31 (0.75, 2.31)	0.34	0.58 (0.26, 1.28)	0.18
Other	0.77 (0.36, 1.63)	0.49	0.39 (0.16, 0.96)	0.04
Health Insurance	--	0.001	--	0.02
Private insurance (ref)	1.00		1.00	
Government Insurance	1.62 (0.96, 2.74)	0.07	2.00 (1.00, 4.03)	0.05
No Health Insurance	2.94 (1.62, 5.33)	<0.001	2.84 (1.36, 5.90)	0.005
Race		0.002		<0.001
White (ref)	1.00		1.00	
African American	2.61 (1.53, 4.45)	<0.001	3.30 (1.77, 6.15)	<0.001
Other	2.42 (1.16, 5.05)	0.019	2.37 (1.06, 5.27)	0.035

**Notes.** COR = crude odds ratio; AOR = adjusted odds ratio. For the regression models, variables that included categories with small cell sizes were recoded as follows. Employment was categorized as: regular full-time work (reference group); regular part-time work; unemployed (either looking or not looking for work); retired; unable to work or disabled; and other (homemaker, student, or other). Health insurance was categorized as: private insurance through participant’s or someone else’s employer or union (reference group); government or other insurance (Medicaid, Medicare, health insurance purchased through federal health insurance marketplace, military programs, or other); and no health insurance coverage. Race was categorized as: white (reference group), African American, or other (Asian, American Indian/Alaska Native, More than one race, other). For the outcome variable of time to first cigarette, 1 = smoke within 5 min of waking (indicating greater nicotine dependence); 0 = smoke first cigarette more than 5 min of waking.

## Data Availability

Data can be accessed from the corresponding author upon reasonable request.

## Abbreviations

The following abbreviations are used in this manuscript:

SES	Socioeconomic Status
CO	Carbon Monoxide
EVPs	Electronic Vapor Products
SD	Standard Deviation
